# Graph-Theory-Based Degree Centrality Combined with Machine Learning Algorithms Can Predict Response to Treatment with Antipsychotic Medications in Patients with First-Episode Schizophrenia

**DOI:** 10.1155/2022/1853002

**Published:** 2022-10-13

**Authors:** Wenming Liu, Peng Fang, Fan Guo, Yuting Qiao, Yuanqiang Zhu, Huaning Wang

**Affiliations:** ^1^Department of Psychiatry, Xijing Hospital, Air Force Medical University, Xi'an, Shaanxi, China; ^2^Department of Military Medical Psychology, Air Force Medical University, Xi'an, Shaanxi, China; ^3^Department of Radiology, Xijing Hospital, Air Force Medical University, Xi'an, Shaanxi, China

## Abstract

**Objectives:**

Schizophrenia (SCZ) is associated with disrupted functional brain connectivity, and antipsychotic medications are the primary and most commonly used treatment for schizophrenia. However, not all patients respond to antipsychotic medications.

**Methods:**

The study is aimed at investigating whether the graph-theory-based degree centrality (DC), derived from resting-state functional MRI (rs-fMRI), can predict the treatment outcomes. rs-fMRI data from 38 SCZ patients were collected and compared with findings from 38 age- and gender-matched healthy controls (HCs). The patients were treated with antipsychotic medications for 16 weeks before undergoing a second rs-fMRI scan. DC data were processed using DPABI and SPM12 software.

**Results:**

SCZ patients at baseline showed increased DC in the frontal and temporal gyrus, anterior cingulate cortex, and precuneus and reduced DC in bilateral subcortical gray matter structures. However, those abnormalities showed a clear renormalization after antipsychotic medication treatments. Support vector machine analysis using leave-one-out cross-validation achieved a correct classification rate of 84.2% (sensitivity 78.9%, specificity 89.5%, and area under the receiver operating characteristic curve (AUC) 0.925) for differentiating effective subjects from ineffective subjects. Brain areas that contributed most to the classification model were mainly located within the bilateral putamen, left inferior frontal gyrus, left middle occipital cortex, bilateral middle frontal gyrus, left cerebellum, left medial frontal gyrus, left inferior temporal gyrus, and left angular. Furthermore, the DC change within the bilateral putamen is negatively correlated with the symptom improvements after treatment.

**Conclusions:**

Our study confirmed that graph-theory-based measures, combined with machine-learning algorithms, can provide crucial insights into pathophysiological mechanisms and the effectiveness of antipsychotic medications.

## 1. Introduction

Schizophrenia (SCZ) is viewed as a disease that involves the dysconnectivity of multiple neuronal circuits [1]. Evidence from resting-state functional magnetic resonance imaging (rs-fMRI) studies has revealed impairments in the interaction within and between large-scale brain networks for SCZ patients [2]. In the context of graph theory [3], disrupted topological organization of the whole-brain network such as reduced functional segregation and enhanced functional integration has been extensively reported in schizophrenia studies, both for functional network and white matter network [4, 5]. Furthermore, reduced degree centrality (DC) of hub regions (brain regions that are connected to a remarkably large number of other regions) is also observed in SCZ [6].

Antipsychotic medications are the primary and most commonly used treatment for schizophrenia. Unfortunately, not all patients respond to antipsychotic medications [7]. Overall estimates suggest that more than 30% of patients have treatment-resistant schizophrenia (TRS) [8]. TRS patients have persistent positive, negative, and cognitive symptoms that lead to poorer outcomes. Previous studies have found functional connectivity impairments within default mode network (DMN) in TRS patients, subcortical networks, and frontal lobes compared with treatment-responsive patients [9]. However, those results are based on average estimates of differences at the group level, and the translational applicability of such data to clinical practice should be based on inferences at the individual rather than group level.

With the recent advancements in the field of machine learning, measurements derived from rs-fMRI combined with artificial intelligence algorithms have led to the improvements in the diagnosis, classification, and treatment outcome prediction for a range of diseases, in particular, for schizophrenia [10]. Our pervious study has indicated that functional connectivity can be a sensitive marker to differentiate SCZ from healthy controls (HCs) [11]; baseline spontaneous regional activities were also found to be predictive of early response to treatment for SCZ [12]. Because SCZ is associated with widespread changes in functional networks, graph-based measurements of network organization, such as degree centrality, might have potential in predicting treatment effects.

The purpose of this study was firstly to determine the dynamic changes of DC measures before and after antipsychotic medication treatment in a group of first-episode SCZ patients. Secondly, we adopt supervised machine learning-based algorithms to investigate whether the baseline DC measures can predict the treatment outcomes. We hypothesized that abnormal DC might reorganize with antipsychotic medication treatment in first-episode SCZ patients and the baseline DC in hub regions of DMN, striatum network, and cerebellum network can accurately classify treatment outcomes.

## 2. Methods

### 2.1. Subjects

Thirty-eight SCZ patients were recruited from the department of Psychiatry, Xijing Hospital affiliated to Air Force Medical University. Clinical diagnosis was according to the Diagnostic and Statistical Manual of Mental Disorders, Fifth Edition (DSM-5). The Positive and Negative Syndrome Scale (PANSS) was assessed by two senior clinical psychiatrists. Subjects were excluded if they have other psychiatric and neurological diseases or any structural abnormalities detected by routine MRI examination. At 16 weeks after first fMRI recording, we performed follow-up assessments of all patients and gathered information about antipsychotic medications and prognosis. In addition, 38 age- and gender-matched healthy controls were recruited by advertisement. This study was approved by the clinical trial ethics committee of Xijing Hospital at the Air Force Medical University. Written informed consent was obtained from each subject prior to the study.

### 2.2. Image Acquisition

The imaging data were collected using a GE 3.0 Tesla Discovery MR scanner with eight–channel phased array head coil (EXCITE, General Electric, Milwaukee, Wisconsin). Subjects were asked to keep their eyes open and to stay awake during the entire session. Using the gradient-echo planar imaging sequence, the resting-state functional images were obtained with the following parameters: echo time = 30 ms, repetition time = 2000 ms, field of view = 240 mm × 240 mm, data matrix = 64 × 64, slices = 33, and total 210 volumes. High-resolution T1-weighted image was also acquired using a volumetric three-dimensional spoiled gradient recall sequence with the following parameters: repetition time = 8.2 ms, echo time = 3.2 ms, field of view = 256 × 256 mm^2^, matrix = 128 × 128, slice thickness = 1 mm, and 196 slices. The same parameters were used for scans of follow-up assessment of SCZ patients and for healthy controls.

### 2.3. fMRI Data Preprocessing

The Data Processing & Analysis for Brain imaging (DPABI, http://rfmri.org/dpabi) was used to preprocess the fMRI data. The first 10 images were removed for magnetization equilibrium; the remaining 200 images were subjected to slice time correction and motion realignment during which the mean frame-wise displacement (FD) was calculated. Data with head motion that exceeded 2 mm and 2° were excluded. Friston-24 model (the 24 parameters include 6 head motion parameters, 6 head motion parameters one time point before, and the 12 corresponding squared items) was used to regress out the effects of nuisance signals and head motions, as suggested by a previous study; global signal averaged over the whole brain was also regressed [13]. Then, the diffeomorphic anatomical registration through the exponentiated Lie algebra (DARTEL) tool was used for normalization (voxel size, 3 × 3 × 3 mm^3^), the normalized data was finally band-pass filtered (0.01–0.08 Hz).

### 2.4. Degree Centrality

A correlation matrix was firstly obtained by computing Pearson correlation coefficients between time courses of each pair of voxels. A threshold of *r* > 0.25 was used to obtain the undirected adjacency matrix in order to remove the weak correlations that might be induced by noise [14]. Then, for each voxel, the degree centrality was calculated as the sum the connections between this voxel with other voxels. DC can be computed as in the following equation:

(1) $$\begin{array}{c} \text{DC}\left(i\right)=\overset{N}{\underset{j=1}{\sum }}a_{ij}, \end{array}$$

where *N* is the number of voxels and *a*_*ij*_ represents the connection or edge from node *i* to node *j*, which is 0 if no edge exists and 1 for an edge with a weight greater than 0.25. For further statistical analysis, the weighted DC was converted into a *z*-score map. Finally, the DC map was smoothed with 6 mm FWHM Gaussian kernel.

### 2.5. Statistical Analysis

Group differences between SCZ patients and HCs in demographic characteristics were compared using the chi-square test and Student's *t*-test with SPSS (IBM SPSS Statistics for Windows, version 19.0, IBM Corp.). For detection of between-group differences in DC, general linear model (GLM) with two-sample *t*-tests (HCs vs. SCZ at baseline; HCs vs. SCZ patients at follow-up) or paired *t*-test (baseline vs. follow-up) was used to identify regional DC changes. The threshold for significance was *P* < 0.05, corrected with the FDR criterion. Age, gender, education, and the mean FD calculated during preprocessing step were accounted by including this term as a covariate. The differences between baseline and follow-up were binarized as a mask for the further machine learning analysis.

### 2.6. Support Vector Machine Analysis

As described in our previous study, SCZ patients were classified as responders or nonresponders according to whether they achieved a reduction in PANSS over 50% [5, 15]. SVM was applied by using the Pattern Recognition for Neuroimaging Toolbox (PRoNTo) (http://www.mlnl.cs.ucl.ac.uk/pronto) to investigate whether the baseline DC can classify antipsychotic medication treatment effects [16]. The SVM soft margin parameter *C* was fixed to its default value 1. Generally, the SVM method includes four steps: (1) feature extraction and feature selection, (2) discriminative region selection, (3) using the training data to train the classifier model, and (4) evaluating the SVM model. In the current study, feature selection consisted of identifying brain regions that differ between the two groups. The above procedure was automatically processed in PRoNTo's “Prepare feature set” programs.

Leave-one-out cross-validation (LOOCV) was used to evaluate the performance of the classifier. In this study, it involved the exclusion of a single subject from each group and training the classifier using the remaining subjects. The above procedures were automatically processed in PRoNTo's “Specify model” programs. Permutation test (1000 times) was used to evaluate the performance of the SVM model; the corresponding accuracy, sensitivity, specificity, and AUC (the area under the receiver operating characteristic curve) were obtained. Then, the weight map was built at voxel level; thus, the region contributions can be ranked and presented for illustration. Finally, the DC changes (baseline vs. follow-up) within these discriminative regions were extracted and Pearson correlation was used to examine the associations between the changes in DC and clinical scores using SPSS. Correction for multiple correlations was accomplished with the FDR criterion.

## 3. Results

### 3.1. Demographic Characteristics

During the follow-up, all patients received second-generation antipsychotic drugs, including paliperidone (10 patients), risperidone (15 patients), olanzapine (9 patients), amisulpride (3 patients), and aripiprazole (1 patient). According to the previous study, drug dose across patients was calculated as olanzapine equivalence. No significant differences were found between patients and HCs on age and gender; detailed demographic characteristics are shown in Table 1.

### 3.2. DC Differences across Groups

**Figure 1:**
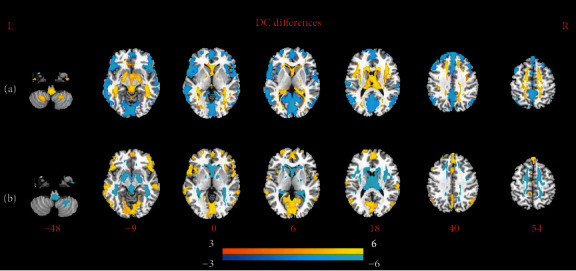
Axial views of significant differences of DC. (a) Regions of increased (warm) and reduced (cool) DC in healthy controls compared with SCZ patients at baseline using two-sample *t* test. (b) Regions of increased (warm) and reduced (cool) DC in children with SCZ patients from baseline to follow-up using paired *t* test.

Compared with healthy controls, significant DC differences were found in SCZ patients at baseline. As shown in Figure 1(a), SCZ patients showed reduced DC in the bilateral cerebellum, putamen, hippocampus, thalamus, and caudate, which are mainly subcortical gray matter structures; SCZ patients showed increased DC in the bilateral inferior frontal gyrus, medial frontal gyrus, superior frontal gyrus, middle temporal gyrus, anterior cingulate cortex, and precuneus. After antipsychotic medication treatment, as shown in Figures 1(b), a restoration of DC within these regions was found. No significant differences were found between SCZ patients at follow-up and healthy controls.

### 3.3. SVM Classification Model

**Figure 2:**
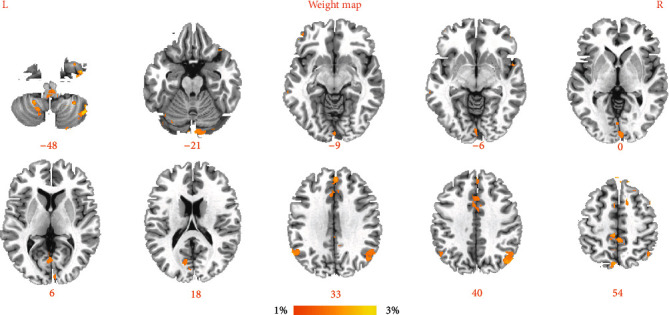
Brain regions of interest that contributed mostly to the accurate classification.

**Figure 3:**
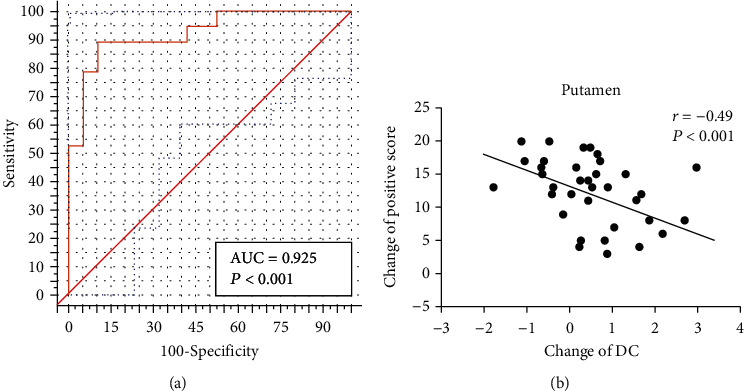
(a) ROC curve of the classifier. (b) Correlation between the clinical symptom improvements and the change of DC values in the bilateral putamen in SCZ patients.

We obtained an accuracy of 84.2% with a sensitivity of 78.9% and specificity of 89.5% for classification of the two groups. As shown in Figure 2(a) and Table 2, the brain regions that contributed most to the classification are listed below; the top 10 regions are the right putamen (discriminative weight 4.51%), left inferior frontal gyrus (discriminative weight 4.21%), left putamen (discriminative weight 4.19%), left middle occipital cortex (discriminative weight 4.13%), left middle frontal gyrus (discriminative weight 3.92%), left cerebellum (discriminative weight 3.81%), left medial frontal gyrus (discriminative weight 3.78%), right middle frontal gyrus (discriminative weight 3.74%), left inferior temporal gyrus (discriminative weight 3.41%), and left angular (discriminative weight 3.41%). As shown in Figure 3(a), the area under the curve for the classification model was 0.925. Finally, to demonstrate the reliability of our results, we also conducted a validation test in a separate sample (10 subjects collected by Siemens MRI). Classification results for the independent validation cohorts were also high (accuracies 0.80), further emphasizing the feasibility of using models trained exclusively on data collected using a different MRI scanner.

### 3.4. Correlation Results

The changes of DC values after antipsychotic medication treatment (baseline-follow-up) were extracted to correlate with the PANSS changes in SCZ patients. Significant negative correlation was found between changes of positive scores and DC value changes in the left putamen (*r* = −0.51, *P* < 0.001) and right putamen (*r* = −0.52, *P* < 0.001). The correlation results are shown in Figure 3(b).

## 4. Discussion

This study explores the abnormalities of DC and antipsychotic medication effect on SCZ patients using resting-state fMRI. Compared with healthy controls, significant DC changes were found for SCZ patients at baseline, but those abnormalities were renormalized after antipsychotic medication administration. Using a multivariate pattern classification method, the present study demonstrates that degree centrality derived from fMRI data collected at baseline can be used to classify subjects on the basis of whether they were effective or ineffective to the antipsychotic medications. With excellent accuracy, the brain regions that showed the most discriminatory power were mainly located within the striatum network, default mode network, and frontal-parietal network. Furthermore, we found a significant negative correlation between the changes of PANSS and DC in the bilateral putamen after treatment. These findings suggest that graph-theory-based measures, such as DC, combined with machine-learning algorithms, can help to predict the effectiveness of antipsychotic medications.

The application of graph theory in neuroimaging allows us to view the human brain as a network at the system level; previous studies have found that patients with first-episode schizophrenia have obvious abnormal network connections [17, 18]. According to a recent study by Jiang et.al, although the cortical thickness was further reduced after antipsychotic medication administration, stronger interregional covariance was found in SCZ patients who showed treatment response. This indicated that the increased network integration induced by second-generation antipsychotic drugs might compensate the disrupted brain structure, which highlights a potential network-level regulatory mechanism of antipsychotics on symptom abnormalities [5]. As a very important indicator of network characteristics, degree centrality should also have obvious abnormalities. Consistent with our hypothesis, we found that under strict FDR test standards, there is a significant difference in the degree centrality between SCZ patients and healthy controls.

Those abnormalities involve almost the entire gray matter area, indicating that there are obvious global and local connection abnormalities in patients with schizophrenia. Compared with task-based functional magnetic resonance imaging studies, resting-state magnetic resonance reduces the deviation caused by the task [19]. This is an important advantage for patients with schizophrenia, as they often have impaired perception and cognitive functions. In addition, functional magnetic resonance data analysis can select regions of interest from a predefined map, which allows the method to be cross-validated between independent populations of subjects. The sensitivity and specificity obtained in this study are equivalent to the classification effect obtained based on structural image magnetic resonance.

We found that the top regions in the classification contribution include the bilateral putamen, which is an important part of the striatum [20]. Molecular imaging studies have found that patients with schizophrenia who respond to drugs have a greater ability to synthesize dopamine in the striatum than patients who do not respond to drugs [21]. Using fMRI [20], White et al. found that the frontal lobe striatum network showed extensive connection damage. More importantly, Sarpal et al. found that the functional connection of the striatum can even predict the response of patients with schizophrenia to drugs [22], which is also consistent with our findings in this study. The abnormality of the striatal network has been extensively confirmed in autopsy reports, structural studies, and functional studies. The frontal-striatal network involves a wide range of functions, including behavioral motivation to cognitive control functions. Dopamine signaling in the striatum also affects the advanced cognitive functions of the prefrontal lobe. Because the negative symptoms of schizophrenia include disorders of cognition, motivation, and social incompatibility, the disordered frontal-striatal connection may be the main cause of negative symptoms. In addition, the frontal-striatal network is also responsible for adaptive behavior, reward learning, and social functions. Disordered function of the striatum, especially the connection between the striatum and the frontal area, may be the main pathway that affects the social perception, decision-making, and emotional performance of patients with schizophrenia.

The top contributing regions also include the left medial frontal lobe, the left inferior temporal gyrus, and the left angular gyrus, which are important areas of the default mode network [23], as well as the left middle frontal gyrus, right middle frontal gyrus, and left occipital middle which are important areas of the frontal-parietal network [24]. The findings in these areas confirm the “triple-network hypothesis” in schizophrenia. This theory has recently been proposed to explain various mental disorders [25]; that is, impaired interactions between these networks may be related to specific psychopathological processes. The default mode network is responsible for self-supervision and is in a negative activation state when performing cognitive tasks; the frontal-parietal network is mainly composed of bilateral dorsolateral prefrontal lobe and the posterior parietal lobe, and it is responsible for attention and working memory functions. The impairment of self-monitoring function often found in patients with schizophrenia may be related to the decline in the internal communication of the default mode network. The disordered activities of the default mode network may also be related to the weakening of the frontal-parietal network.

Our research has the following limitations. First of all, in the baseline state, the patient population received antipsychotic treatment. Although the patients were only exposed to antipsychotics within 2 weeks, the potential impact of the drug's contribution to the results cannot be ignored; another limitation is that all patients received second-generation antipsychotic drugs, but each patient's sensitivity to the drug is heterogeneous, and such heterogeneity will also have an impact on the current research results; finally, our study only recruited 38 cases of SZ patients and 38 healthy controls, the sample size of the validation cohorts was also too small, the statistical power may be limited, and further study should increase the sample size to guarantee the statistical power.

## 5. Conclusion

We showed a normalization of the degree centrality in patients with SCZ after antipsychotic medication treatments. With the help of magnetic resonance imaging and machine learning algorithms, the characteristics of brain networks in schizophrenia can be used as sensitive biomarkers for treatment prediction of SZ.

## Figures and Tables

**Table 1:** Demographic characteristics and clinical measures of schizophrenia patients and healthy controls.

	SZ (*n* = 38)	HCs (*n* = 38)	*t*/*χ*^2^	*P* value
Demographic characteristics				
Age (y)	16-59 (24.1 ± 8.6)	17-58 (24.5 ± 8.3)	-0.2	0.82
Gender (male/female)	21/17	21/17	0	1^b^
Education (y)	4-19 (12.2 ± 3.1)	7-20 (14.1 ± 3.0)	-2.7	0.01^∗^
Clinical measurements				
Family history (y/n)	5/33	—	—	—
Duration (y)	0.02-8 (1.29 ± 1.93)	—	—	—
Treatment before (d)	0-13 (4.62 ± 3.95)	—	—	—
Interscan interval (m)	1-12 (4.6 ± 1.9)	—	—	—
Olanzapine (mg/d)	3.7-27.3 (15.71 ± 4.91)			
PANSS baseline				
Positive score	7-31 (22.7 ± 4.7)	—	—	—
Negative score	7-34 (21.3 ± 6.4)	—	—	—
General score	30-64 (44.5 ± 8.4)	—	—	—
Total score	63-124 (88.9 ± 13.1)	—	—	—
PANSS follow-up				
Positive score	7-21 (9.7 ± 3.7)	—	—	—
Negative score	7-28 (14.9 ± 5.2)	—	—	—
General score	11-42 (25.3 ± 5.8)	—	—	—
Total score	30-110 (53.6 ± 14.1)	—	—	—

Data were presented as mean ± SD. Abbreviation: SD: standard deviation; PANSS: positive and negative syndrome scale; ^∗^*P*: *T*-test with *P* < 0.05.

**Table 2:** The top ten brain regions that contributed mostly to the accurate classification.

Brain regions	Cluster size	Peak coordinates (MNI)			Discriminative weight (%)
		*X*	*Y*	*Z*	
Right putamen	354	12	0	6	4.51
Left inferior frontal gyrus	68	-48	27	18	4.21
Left putamen	190	-21	-6	-9	4.19
Left middle occipital gyrus	79	-24	-99	-6	4.13
Left middle frontal gyrus	114	-21	6	54	3.92
Left cerebellum	179	-51	-57	-48	3.81
Left medial frontal gyrus	91	-6	63	6	3.78
Right middle frontal gyrus	61	33	9	54	3.74
Left inferior temporal gyrus	54	-63	-21	-21	3.41
Left angular	73	-39	-51	33	3.41

## Data Availability

The patient data used to support the findings of this study are restricted by the Institutional Review Board of the Xijing Hospital in order to protect patient privacy.
